# The Bioaccumulation and Tissue Distribution of Arsenic Species in Tilapia

**DOI:** 10.3390/ijerph16050757

**Published:** 2019-03-02

**Authors:** Jia Pei, Jinxing Zuo, Xiaoyan Wang, Jingyu Yin, Liping Liu, Wenhong Fan

**Affiliations:** 1 School of Space and Environment, Beihang University, Beijing 100191, China; peijia2011@163.com (J.P.); zuojinxing35@163.com (J.Z.); wangxy_926@buaa.edu.cn (X.W.); qdyinjingyu@163.com (J.Y.); 2 Beijing Advanced Innovation Center for Big Data-Based Precision Medicine, Beihang University, Beijing 100191, China; 3 Beijing Key Laboratory of Diagnostic and Traceability Technologies for Food Poisoning, Center for Disease Prevention and Control, Beijing 100191, China; llp9312@163.com

**Keywords:** arsenic, tissue distribution, bioaccumulation, tilapia, risk assessment

## Abstract

Arsenic is a public concern due to its widespread occurrence and carcinogenicity. Consumption of arsenic-contaminated fish is an important exposure pathway for human health. This study focused on understanding how exposure to arsenic-contaminated fish is informative to human health risk assessment. While the bioaccumulation and tissue distributions of total arsenic concentration in fish are commonly reported, there are limited studies related to the time-course of arsenic species in various tissues. Using the Tilapia as a case, this study aimed to investigate the bioaccumulation and tissue distributions (liver, gastrointestinal (GI), muscle, and gill) of arsenic species in freshwater fish via diet-borne inorganic arsenic exposure. In particular, the Tilapia were exposed to arsenic (III) and As(V) for 32 days. The accumulation of arsenic in all tissues linearly increased with time in the first 10 days’ exposure, while the arsenic levels remained stable in the following 20 days’ exposure. The accumulation of arsenic in tissue followed the sequence of intestine > liver > gill > muscle. Meanwhile, more than 90% of arsenic was converted into organic form in liver, gill, and muscle, while organic arsenic contributed about 30–80% to the total arsenic in the GI. The percentage of organic form in muscle is the highest, followed by gill, liver, and intestine, and arsenobetaine is the main form of organic arsenic. While the exposure profiles of As(III) and As(V) are quite similar, the absorption rate of As(V) is relatively higher than that of As(III). Information provided here can be instrumental for exposure assessment and risk management for arsenic in aquatic environment.

## 1. Introduction

Arsenic is a public concern due to its high toxicity and widespread occurrence [1]. In China, approximately 19.6 million people were at the risk of being exposed to arsenic-contaminated groundwater [2]. The fish can be cultivated in groundwater leading to humans being exposed to arsenic via diet and water, as exemplified arsenic levels in fish can be up to 110 μg/g in some contaminated areas [3,4]. Therefore, by the consumption of fish, arsenic is subsequently accumulated in human body. As acknowledged, the toxicity of arsenic not only depends on its concentration, but also on its speciation [5]. Inorganic arsenic, a highly toxic compound, is a carcinogen recognized by the international agency for research on cancer (IARC), and organic arsenic is much less toxic [6]. The total arsenic is reported within a range of 0.2~150 μg/g for marine fish and bioaccumulation varied for different tissues. More than 90% of arsenic is of organic form with less toxicity, while the content of inorganic arsenic is only 0.02%~11% in marine fish [7,8]. It is reported that the As(V) taken up by marine fish can be first transformed into As(III), and then As(III) is easily converted to monomethylarsonic acid (MMA), dimethylarsinic acid (DMA) and/or arsenobetaine (AsB), which is the final speciation of arsenic [9,10]. 

The tissue distributions of arsenic in fish were reported in previous studies. For example, studies indicated the high accumulation of arsenic in most tissues occurred in polluted areas [11,12]: when brown trout (*Salmo trutta*) were exposed in sediments of 216.75 μg/g and water of 43.07 μg/L, results showed operculum, liver, and gills have higher As concentrations, whereas As levels in muscle were approximate three folds lower [13]. However, most related studies only reported bioaccumulation and tissue distributions of total arsenic concentration in fish; studies focusing on the time-course of arsenic species in tissues are relatively rarer. For example, the majority (>50%) of organic arsenic in almost all tissues from five freshwater fish species caught in Back Bay near Yellowknife was not directly identified [14], and thus the tissue distribution of arsenic species remains unclear. Some studies also show that freshwater fish have a strong ability to convert inorganic arsenic to organic arsenic, and thus the AsB level is relatively high, although the proportion of AsB varied among different fish [15,16]. 

Thus, solidifying our knowledge of arsenic behavior requires more understanding on the absorption, distribution, metabolism, and excretion of arsenic species in the aquatic environment. Tilapia is rich in protein and essential fatty acids, which plays an important role in the inland human diet [17]. Using the Tilapia as a case study, we attempted to observe the bioaccumulation and tissue distribution of arsenic species in freshwater fish via diet-borne inorganic arsenic (both As(III) and As(V)) exposure. Based on a sub-chronic exposure experiment, the specific aims of this study include: (1) to report the time-course of total arsenic exposure and arsenic species in most fish tissues, such as gastrointestinal (GI), liver, gill, and muscle; (2) to address the tissue distribution of arsenic species during the exposure duration after inorganic arsenic exposure; (3) to compare the accumulation differences between As(III) and As(V) exposure. Information provided here should be instrumental for exposure assessment and risk management for arsenic in the freshwater environment.

## 2. Materials and Methods

### 2.1. Diets and Experimental Design

Tilapia (*Oreochromis spp*) were obtained from a fish farm in Beijing, China. They were maintained in natural freshwater (25 °C) and fed with artificial diets twice a day at 3% of their body weight. They were acclimated to the test conditions for 2 weeks prior to exposure experiment. Feces and uneaten food were removed twice a day. 

The artificial diets were purchased from a feed company in Beijing, China. The diets were labeled with As(III) and As(V) as an aqueous solution of arsenite and arsenate (NaAsO_2_ and Na_2_HAsO_4_·7H_2_O, Sigma, USA), respectively, to achieve a nominal concentration of 700 μg/g diet. When the diet pellets were completely soaked within the As solution, they were dried at 60 °C for 1~2 h to constant weight. The diets were then stored at −20 °C in sealed polyethylene bags until they were used. 

264 Tilapia were exposed to diet-borne As treatments (88 Tilapia in each treatment (As(III), As(V), and control)). Fish were fed a certain amount of artificial food once a day, then changing the water after feeding for two hours to prevent waterborne As exposure and collecting the Tilapia’s excrement every day. Six Tilapia in each treatment and their intestine, liver, gill, and muscle tissues were carefully dissected and collected under low temperature on 2 d, 4 d, 6 d, 8 d, 10 d, 13 d, 16 d, 20 d, 24 d, 28 d, and 32 d of exposure, respectively. The standard length and wet weight of each individual fish was immediately measured. Then they were frozen-dried (freeze drier), homogenized (grinding), and stored in small polyethylene tubes at −80 °C for total As and As speciation analysis. 

### 2.2. Total Arsenic Concentrations

About 0.05~0.15 g of samples were weighed in a 10mL digestion tube and digested with 2 mL of concentrated HNO_3_ (65%, analytical reagent grade) in digest instrument at 120 °C for 20 h until clarification. After cooling, the samples were diluted to 5 mL with Milli-Q water. A blank digest was processed by following the same procedure. The total As was analyzed using inductively coupled plasma mass spectrometer (ICP-MS, Thermo Fisher icap Q series). The standard solution was prepared by serial dilution from a stock solution (National China Standard, National Institute of Metrology, China). The accuracy of the digestion method was testified by analyzing the standard reference materials (SRM) BCR-627 Tune Fish Tissue (Institute for Reference Materials and Measurements, Geel, Belgium). The detected total As concentration in tuna fish was 4.89 ± 0.12 μg/g, compared to the certified value of 4.81 ± 0.3 μg/g. The artificial diets as well as collected excrement were also digested and the total As concentrations were simultaneously measured. The total As recovery rate of SRM was 94%, and the As concentrations were expressed in μg/g dry weight.

### 2.3. The Determination of Speciation 

All solutions were prepared with double-distilled water (ddH_2_O). The standard solution was prepared by serial dilution from different stock solutions including As(III), As(V), AsB, MMA and DMA (National China Standard, National Institute of Metrology, China) for arsenic speciation analysis. Mobile phases in liquid phase chromatography (LC) is 7 mmol NH_4_H_2_PO_4_ and pH is adjusted to 8.5 by 10% NH_3_H_2_O. 10% (V:V) HCl and 20 g/L KBH_4_ employed for hydride generations. 10 g/L K_2_S_2_O_8_ was used in the photo-oxidation reaction. The daily prepared KBH_4_ solution was not filtered before use. Samples were filtered through a 0.45 μm polytetrafluoroethylene (PTFE) membrane (China). The standard solutions were stored in high-density polypropylene containers at 4 °C. Analytical working standards were prepared daily by diluting the stock solutions with ddH_2_O prior to analysis. The freeze-dried fish tissues were prepared for As speciation analysis using methanol/water (1:1 v/v) extraction. 

About 0.05 g of sample was accurately weighed and transferred into 10 mL centrifuge tubes with 5 mL of 50% methanol solution. The mixtures were homogenized with a tissue homogenizer for 15 min, then centrifuged at 9000 rpm for 10 min and the supernatant then poured into 10 mL centrifuge tubes. This extraction process was repeated twice with the supernatant being added to the previous extract. The final extraction (a combination of the two supernatants approximately 10 mL in total) was heated to 50 °C to evaporate the solvent until a volume of approximately 1 mL was reached. The concentrated samples were then diluted with ddH_2_O to a volume of 2 mL. Samples were filtered through 0.45 μm syringe filters into 5 mL centrifuge tube in the preparation for LC-UV-HG-AFS (LC-AFS 9531 Beijing, China) analysis. 

**Figure 1:**
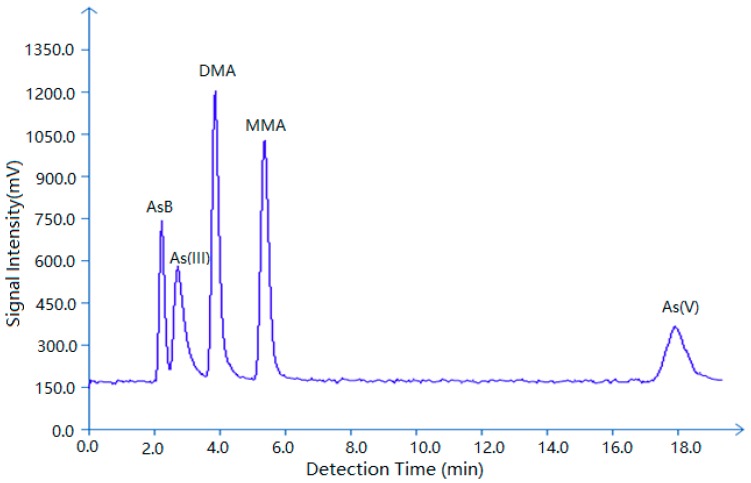
The detection time and signal intensity for arsenic species in standard reference material (80 μg/L AsB, As(III), DMA, MMA, and As(V) mix solution).

The extracted samples (100 μL) were injected into the chromatographic column used for As species separation. The extraction efficiencies and analysis methods were evaluated by the analysis of standard reference material tuna fish (BCR-627 Belgium). BCR-627 tuna fish tissue (0.1 g) was used for AsB and DMA analyses. The BCR-627 reference material contained AsB 3.28 ± 0.26 μg/g (84.19% recovery of 3.90 ± 0.22 μg/g certified value, n = 6) and DMA 0.097 ± 0.0067 μg/g (65.22% recovery of 0.15 ± 0.002 μg/g certified value, n = 6). Spikes were used to confirm the recovery of other As species detected during speciation analysis. The detection time for arsenic species was plotted in Figure 1. In our study, the recoveries of As(III), As(V) and MMA were 65.41%, 77.00%, and 76.81%, respectively. The detection limitations for As(III), As(V), MMA, DMA, AsB, and total Arsenic were 0.25, 0.5, 0.25, 0.25, 0.5, and 0.1 ppb, respectively. 

## 3. Results and Discussion

### 3.1. The Concentration and Speciation of Arsenic in Diet and Excrement

The arsenic concentrations were 806.5 and 772.1 µg/g (dry weight) in As(III) and As(V) artificial diets, respectively. In particular, the As(III) and As(V) accounted for 16.63% and 83.37% respectively in Artificial diets labeled As(V), while the corresponding ratios were 83.28% and 16.72% in Artificial diets labeled As(III).

No mortalities of Tilapia were observed through the experiment period. After 32 days’ exposure, there is no significant difference in body length and weight of Tilapia between the blank groups and the As(III, V) food exposure groups. The arsenic concentration in blank group in Tilapia feces was 1.200.41 µg/g, while the arsenic in excrement was 83.4017.96 µg/g and 133.2384.64 µg/g for the Tilapia exposed by As(III) and As(V). The speciation in excreta is mainly inorganic arsenic, which was up to 85%. By contrast, the dimethyl arsenic and arsenic betaine were not detected in all excrements. This indicated most unabsorbed arsenic was from GI tract, rather than from the biliary excretion. 

### 3.2. Total Arsenic in Different Tissues

**Figure 2:**
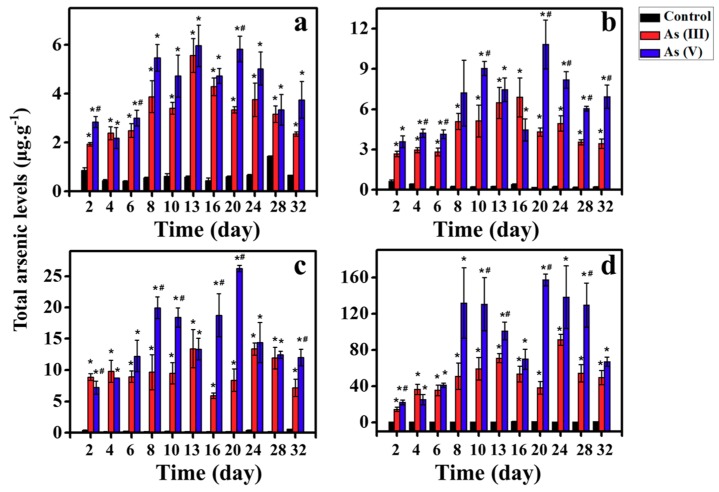
The time-course of total arsenic concentrations in Tilapia: (**a**) muscle; (**b**) gill; (**c**) liver; and (**d**) GI. Asterisk (*) indicates significant differences between total As concentration in experimental and control groups (*p* < 0.05), while hash (#) represents significant differences between total As concentration in experimental As(III) groups and As(V) groups (*p* < 0.05).

The total As concentrations in liver, GI, muscle, and gill were all lower than 1.5 µg/g with a range of 0.15–1.44 in blank groups as illustrated in Figure 2. Also, Figure 2 summarizes the time-course concentrations in different tissues in Tilapia exposed to As(III). Significant differences (*t*-test) were observed in total arsenic between measured control groups and experimental groups as mapped in Figure 2. As illustrated, the accumulation of total arsenic in the GI is the highest, followed by liver, gill, and muscle. The concentrations in all tissues increased sharply during the initial 10 days’ exposure, and then reached a plateau. In particular, after the initial 10 days’ exposure, the total arsenic for As(III) exposure is 59.21 ± 12.45 μg/g in GI, 9.51 ± 1.68 μg/g in liver, 5.12 ± 0.61 μg/g in gill and 3.40 ± 0.24 μg/g in muscle. There are positive correlations between arsenic accumulation and exposure days during the initial exposure 10 days (R^2^ > 0.65 *p* < 0.05), suggesting the accumulation of arsenic increased linearly with time for continuing food exposure. The daily accumulation rate for GI was the highest, which was up to 5.75 μg/g/day. During the following 22 days of exposure, the mean concentrations in the GI, liver, gill, and muscle were 59.53 ± 17.07 μg/g, 10.04 ± 2.99 μg/g, 4.94 ± 4.62 μg/g and 3.74 ± 3.38 μg/g, which were comparable to concentrations on the 10th exposure day. These comparable concentrations indicate the arsenic levels in Tilapia can reach a stable status after the 10th day of exposure. In previous studies, when herbivorous fish *Siganus fuscescens* were exposed to 1500 μg/g diet-borne for 21 d, the concentration of As are 3.11 μg/g, 3.39 μg/g, and 2.63 μg/g in GI, liver, and muscle, respectively, which were relatively lower than those in Tilapia. 

Similarly, for As(V) exposure, the arsenic levels in tissues linearly increased with the exposure time during the initial 10 days’ exposure (Figure 2). On the 10th day of exposure, the accumulation of arsenic for As(V) exposure group was 130.49 ± 29.19 μg/g in GI, which was 120.37% higher than the corresponding concentration based on As(III) exposure. The total arsenic concentration in gill, liver, and muscle were determined to be 9.04 ± 0.53 μg/g, 18.40 ± 1.57 μg/g, and 4.72 ± 0.86 μg/g on 10d, respectively. The total arsenic concentration in the liver, gill, and muscle were all significantly higher (paired *t*-test, *p* < 0.05) than those based on As(III) exposure (Figure 2), while the ratios were calculated with a range of 38.71%–93.56%. Obviously, the accumulation rate for As(V) exposure is accordingly higher than that for As(III) exposure. Also, for As(V) exposure, the arsenic levels in tissues were stable during the exposure period of 10–32 days, with the 110.45 ± 37.56 μg/g in GI, 16.20 ± 5.47 μg/g in liver, 7.32 ± 2.14 μg/g in gill, and 4.77 ± 1.07 μg/g in muscle. 

Previous studies have reported the intestinal environment of Tilapia is acid, and As(III) is difficult to disintegrate under pH 4~8, and thus As(III) came into cells in the form of neutral particles through the water channel protein [18]. For As(V), its acidity coefficient (pk_a_) value is 2.2 and 6.97, and therefore $H_{2}{\mathrm{AsO}}_{4}^{-}$ and ${\mathrm{HAsO}}_{3}^{-}$ could enter cells by the phosphate transport corridor that commonly exists in the gut environment. At the same time, due to the high affinity between As(Ⅲ) and mercaptan, it is easy to combine with glutathione (GSH) and cysteine that exist in liver or blood to generate compounds that can be ultimately excreted through the urine. Compared to As(III), As(V) has a week affinity with mercaptan and it may reduce to As(III) by GSH/CySH after As(V) enters Tilapia [19]. As(III) is more easily combined in the blood to excrete in vitro. Therefore, the accumulation rate of As(V) exposure in living organisms is higher than that of As(III) exposure. On the other hand, the common way of arsenic detoxification in Tilapia is extracellular or isolation in organelles, and it is easier to excrete As(III) than As(V) by Tilapia. These possible reasons result in the significantly higher arsenic levels due to As(V) exposure. 

Once ingested by fish, As is absorbed from GI tract and enters circulation. Arsenic rapidly leaves the blood and is distributed to other tissues [20]. Previous studies have found that in freshwater ecosystems, the content of arsenic in the liver is higher than that of muscles [20]. This may be explained by noting that the muscle does not have direct contact with the arsenic and the muscle is not active part of detoxification [12,21]. The study on toxicity of freshwater fish has shown that arsenic is absorbed mainly through gills in the aqueous phase, and thus the accumulation is higher in the gills [22]. However, in our study, due to the concentration of arsenic in water, this phase is lower by about 10 μg/g, mainly through digesting to get arsenic, and gills directly obtained from the water accumulation is less. 

### 3.3. Tissue Distribution of Arsenic Species 

The following five As species were measured in all tissues: As(III), As(V), MMA, DMA, and AsB. The percentages of organic As in different tissues due to inorganic arsenic exposure were tabulated in Table 1 and Table 2. Obviously, the percentages of organic arsenic were quite similar between As(III) and As(V) exposure. In the exposure duration, the proportion of organic arsenic increased sharply during the initial exposure days, which suggested the inorganic arsenic can be rapidly converted to organic arsenic in the liver or elsewhere. 

The proportion of organic arsenic in the muscle was the highest, followed by the gills, liver, and GI, and the percentages of organic arsenic were commonly more than 90% in tissues. It is obvious the organic arsenic was transported to the body of Tilapia by blood, after the methylation of arsenic in the liver, or elsewhere. Compared to the gill and muscle, the lower ratio of organic arsenic in liver may be caused by portal circulation. Meanwhile, the inorganic rather than organic arsenic was the major compositions in GI and the percentages of DMA and AsB were relatively lower (Table 1 and Table 2). Previous study also indicated that the proportion of organic arsenic in GI was lower than that in the liver, muscle, and gill [23]. This phenomenon may be caused by the low levels of methylation in the gut than the liver, muscles, and gills [24]. Also, the GI tract is the first step of arsenic biotransformation, and GI is directly exposed to inorganic arsenic via diet. 

**Figure 3:**
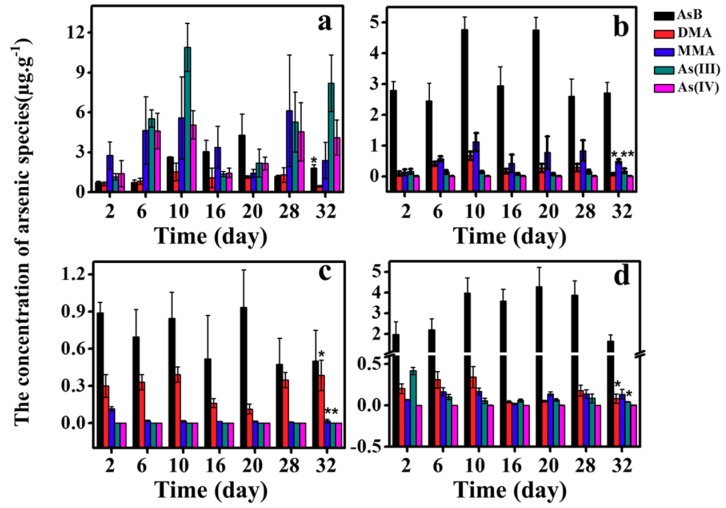
The concentrations of five As species (AsB, DMA, MMA, AS(III), and As(IV)) in: (**a**) intestine; (**b**) liver; (**c**) muscle; and (**d**) gill over 32 days of As(III) exposure. Analysis of variance was used to test differences of arsenic species during the experiment period and significance was marked on 32nd day (*p* < 0.05).

**Figure 4:**
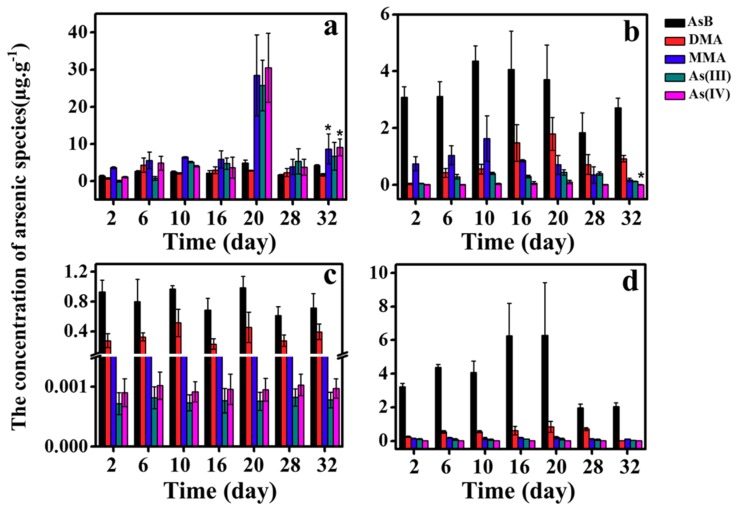
The concentrations of five As species (AsB, DMA, MMA, AS(III) and As(IV)) in: (**a**) intestine; (**b**) liver; (**c**) muscle; and (**d**) gill during 32 days of As(V) exposure. Analysis of variance was used to test differences of arsenic species during experiment period and significance was marked on 32nd day (*p* < 0.05).

Figure 3 and Figure 4 present the concentrations of five As species in each tissue during the exposure period. Generally speaking, the arsenic species distribution for the four tissues is similar between As(III) and As(V) exposure. Although it seems that the concentrations of arsenic during latter experimental period are higher than early experimental period, results from analysis of variance (ANOVA) indicated significant differences were observed for limited cases. This phenomenon can be explained by stable concentrations in days 10–32 and high variance in most experimental groups. Using As(III) exposure as an example, the concentrations of AsB increased from 2 to 5 μg/g and accounted for over 70% in liver, and both DMA and MMA also increased gradually with the time. In gill, AsB is the main form of arsenic, which accounts for 75% of total arsenic. Meanwhile, the concentration of DMA increased from 0.04 μg/g to 0.48 μg/g, while MMA and inorganic arsenic did not show obvious increase. In muscle, AsB levels were detected with a range of 0.5~1.2 μg/g. The AsB and DMA increased with exposure time and the sum of both proportions is close to 95%. Overall, AsB was the main form of arsenic in muscle, gill, and liver, while the proportion of AsB in the GI is relatively lower. 

**Figure 5:**
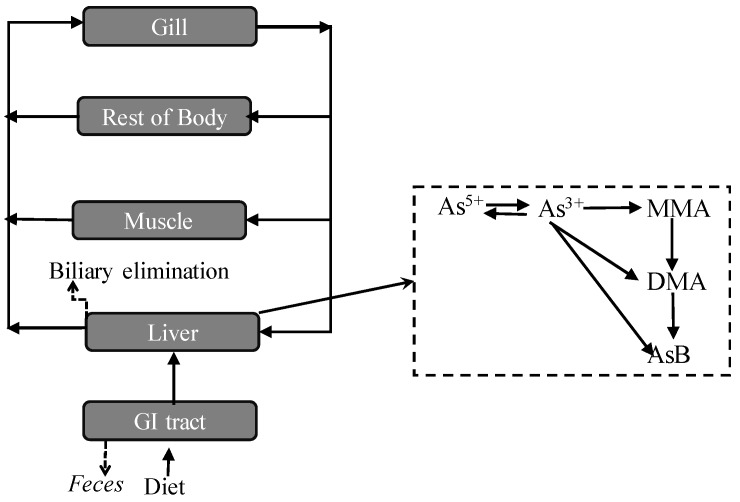
The absorption, distribution, metabolism, and excretion of inorganic arsenic exposure in Tilapia.

The absorption, distribution, metabolism, and excretion of inorganic arsenic exposure is important to human health risk assessment [25]. In this study, the behavior of inorganic exposure in Tilapia can be described by Figure 5. Based on experimental data (using the accumulation of total arsenic in GI), the absorption rate of As(V) is approximately 120% higher than that of As(III). The inorganic arsenic is firstly oxidized, reduced, and methylated in GI, while the ratio of methylation is lower than that in liver. As acknowledged, As(III) can be oxidized to As(V) and As(V) can be reduced to As(III), and the conversion between As(III) and As(V) also occurred in the GI tract of Tilapia. Similar results showed that As(V) reduction and As(III) oxidization exist in algae [26]. As(V) reduction and methylation are usually considered a detoxification path. In GI, inorganic arsenic can also be transformed into MMA, and excrement that contains large amounts of inorganic arsenic (about 80%) and MMA (about 20%) are out of the body. Thus, only a small fraction arsenic was absorbed by GI tract and further methylated to produce DMA and AsB, which are not easily excluded from the body. Arsenic reached the liver with the blood circulation, which is the main site to methylate, and the proportion of DMA and AsB are increased. Compared to the ratio between MMA and DMA, DMA is a more stable form in Tilapia. 

As previously discussed, the AsB levels in tissues of Tilapia increased with the time (Figure 3 and Figure 4). AsB is synthesized continually in Tilapia and distributed to different tissues. Many studies have shown that AsB is the final product of arsenic in Marine organisms [10,27]. In some freshwater organisms, there is also bio-transform between inorganic arsenic and AsB, and transformation from iAs to AsB was observed in freshwater plankton organisms collected from contaminated lakes [28]. The highest proportion of AsB can be up to 70% in gill, and the AsB may be retained and accumulated by the organism in water-stressed environments to serve as an osmolyte instead of glycine-betaine in gill [29]. The synthesis path of AsB in freshwater fish is not clear, but in our study there may be two paths to generate AsB: a) DMA is precursor of AsB; or b) arsenic sugar is produced in freshwater fish first, and then arsenic sugar is easily degraded in organisms, and further oxidized or methylated to produce AsB [30,31]. 

Given that Tilapia constitute important food for inland people, it is crucial to investigate the arsenic behavior in Tilapia. Compared to marine fish, 1.28~22.8%, the inorganic arsenic has a lower proportion in muscle, liver, and gill in Tilapia, at just 0.35%~5%. In previous studies, the sequence of As species in marine fish organisms was AsB > DMA > MMA≈As(V) and As(III) in 19 marine organisms, which indicated low health risks considering organic arsenic is much less toxic [32]. The findings in our study showed the similar sequence of As species. Also, in our study, more than 50% of organic arsenic has not been detected, which may be converted into arsenic sugar, and show low levels of harm to human health. 

## 4. Conclusions

This study reported the bioaccumulation and tissue distributions of arsenic species based on a 32-day exposure in Tilapia. The major findings derived from this sub-chronic experiment include: (1) the accumulation of total arsenic in fish tissues linearly increases with exposure time in initial exposure period (approximately 0–10 days), and then reaches a plateau. The accumulation of arsenic is intestine > liver > gill > muscle, which may be explained by the arsenic behavior in Tilapia. In detail, after exposure to inorganic exposure, the inorganic arsenic is absorbed in the GI, and majorly metabolized in liver. Then, the arsenic was transported to the body of fish via blood circulation. (2) The ratio of organic form in the muscle is the highest, followed by gill, liver, and GI, and AsB, the final production of arsenic metabolism, is the main form of organic arsenic. The lowest organic form in GI is caused by low metabolism in GI, and GI is directly exposed to inorganic As. In particular, more than 90% of arsenic was observed in organic speciation in liver, gill, and muscle. (3) While the exposure profile, such as tissue distribution and bioaccumulation, between As(III) and As(V) is similar, the absorption rate of As(V) is relatively higher than that of As(III), which is exemplified by the fact that arsenic concentrations in tissues after exposure of As(V) are higher than their counterparts from As(III) exposure. 

## Figures and Tables

**Table 1:** The percentage of organic arsenic in different tissues for As(III) exposure.

Exposure Time (days)	GI (%)	Liver (%)	Gill (%)	Muscle (%)
2	70.30 ± 7.84	95.38 ± 8.78	83.18 ± 5.08	99.30 ± 6.17
4	41.15 ± 7.65	97.05 ± 5.38	96.58 ± 0.41	99.86 ± 5.42
6	43.42 ± 6.65	94.43 ± 6.01	96.31 ± 3.13	99.83 ± 4.59
8	62.91 ± 9.71	97.84 ± 4.77	98.84 ± 4.60	99.91 ± 7.56
10	43.28 ± 4.66	91.10 ± 9.64	99.00 ± 1.20	99.83 ± 3.92
13	60.45 ± 16.20	97.30 ± 9.11	98.87 ± 4.78	99.90 ± 8.02
16	52.29 ± 9.08	96.19 ± 10.59	98.39 ± 1.13	99.66 ± 10.08
20	63.27 ± 20.99	98.73 ± 9.77	98.67 ± 1.79	99.85 ± 8.01
24	48.02 ± 13.48	96.97 ± 11.11	98.48 ± 11.49	99.79 ± 10.78
28	51.93 ± 9.86	93.48 ± 11.17	98.48 ± 9.06	99.80 ± 14.05
32	30.53 ± 6.32	87.58 ± 16.78	98.39 ± 13.31	99.79 ± 16.08

**Table 2:** The percentage of organic arsenic in different tissues for As(V) exposure.

Exposure Time (days)	GI (%)	Liver (%)	Gill (%)	Muscle (%)
2	83.93 ± 8.38	96.86 ± 7.21	96.15 ± 9.94	99.89 ± 12.29
4	63.59 ± 4.31	96.64 ± 7.69	99.49 ± 16.81	99.90 ± 2.96
6	62.45 ± 6.19	88.68 ± 6.88	97.39 ± 4.35	99.84 ± 3.25
8	58.46 ± 9.78	92.91 ± 3.96	95.18 ± 16.98	99.71 ± 4.70
10	54.79 ± 1.03	80.83 ± 2.08	94.51 ± 5.40	99.85 ± 4.25
13	69.67 ± 2.02	97.66 ± 3.26	94.71 ± 8.65	99.94 ± 4.52
16	52.04 ± 3.13	90.03 ± 12.59	98.94 ± 9.58	99.29 ± 20.08
20	37.58 ± 9.09	91.39 ± 8.74	94.71 ± 7.07	99.89 ± 19.43
24	38.27 ± 7.53	87.42 ± 6.18	97.52 ± 7.29	99.86 ± 4.80
28	45.83 ± 9.84	86.86 ± 8.46	94.96 ± 5.96	99.78 ± 4.48
32	42.70 ± 6.31	96.94 ± 16.39	99.79 ± 0.93	99.85 ± 20.25
